# Selection of ovine housekeeping genes for normalisation by real-time RT-PCR; analysis of *PrP *gene expression and genetic susceptibility to scrapie

**DOI:** 10.1186/1746-6148-1-3

**Published:** 2005-09-28

**Authors:** David Garcia-Crespo, Ramón A Juste, Ana Hurtado

**Affiliations:** 1 Department of Animal Health, Instituto Vasco de Investigación y Desarrollo Agrario (NEIKER); Berreaga, 1. 48160 Derio, Bizkaia, Spain

## Abstract

**Background:**

Cellular prion protein expression is essential for the development of transmissible spongiform encephalopathies (TSEs), and in sheep, genetic susceptibility to scrapie has been associated to *PrP *gene polymorphisms. To test the hypothetical linkage between *PrP *gene expression and genetic susceptibility, *PrP *mRNA levels were measured by real-time RT-PCR in six ovine tissues of animals with different genotypes.

**Results:**

Previous to the *PrP *gene expression analysis the stability of several housekeeping (HK) genes was assessed in order to select the best ones for relative quantification. The normalisation of gene expression was carried out using a minimum of three HK genes in order to detect small expression differences more accurately than using a single control gene. The expression stability analysis of six HK genes showed a large tissue-associated variation reflecting the existence of tissue-specific factors. Thereby, a specific set of HK genes was required for an accurate normalisation of the *PrP *gene expression within each tissue. Statistical differences in the normalised *PrP *mRNA levels were found among the tissues, obtaining the highest expression level in obex, followed by ileum, lymph node, spleen, cerebellum and cerebrum. A tendency towards increased *PrP *mRNA levels and genetic susceptibility was observed in central nervous system. However, the results did not support the hypothesis that *PrP *mRNA levels vary between genotypes.

**Conclusion:**

The results on *PrP *gene expression presented here provide valuable baseline data for future studies on scrapie pathogenesis. On the other hand, the results on stability data of several HK genes reported in this study could prove very useful in other gene expression studies carried out in these relevant ovine tissues.

## Background

Scrapie is a neurodegenerative disease of the group of transmissible spongiform encephalopathies (TSEs) that affects sheep and goats [1,2]. The lesions appear mainly in the nervous system in the form of vacuoles triggered by the conversion of the cellular prion protein (PrP^c^) into the abnormal isoform (PrP^Sc^) followed by its pathological accumulation [2]. Although the exact origin of the disease remains unknown, the "protein only" hypothesis supports the concept that PrP^Sc ^is the transmissible agent causing the disease [2]. Therefore, the presence of PrP^c ^is essential to develop scrapie [3,4]. However, little is known about the physiological role of PrP^c ^and basic PrP^c ^regulating mechanisms.

The development of clinical signs of scrapie has been linked to some *PrP *gene polymorphisms [5,6]. According to this genetic profile, animals have been classified into risk groups from Type 1 to Type 5 in increasing order of susceptibility to scrapie according to the UK National Scrapie Plan (NSP) [7]. The reasons for the different genetic susceptibility have been assessed *in vitro *revealing low conversion efficiencies of PrP^c ^into PrP^Sc ^in resistant genotypes [8,9]. However, individual factors, and unknown genes or proteins might be involved in this genetic susceptibility.

The oral route is the main pathway of transmission of prions in nature. Once the agent has entered into the host, an early amplification occurs in the lymphoreticular system followed by the subsequent spread to several tissues through lymphatic routes, blood or peripheral nervous system. This precedes replication in the central nervous system (CNS) [10,11]. Taking into account that the presence of PrP^c ^is essential to develop scrapie [3,4], knowing the distribution of PrP^c ^in lymphoid and nervous tissues is relevant to understanding the pathogenesis of the disease. Likewise, *PrP *transcript levels and subsequent translation product abundance might play an important role in the transmission and development of the disease. In this sense, absolute quantification studies on bovine and golden hamster *PrP *gene expression revealed high levels of *PrP *mRNA in CNS and lymphoid tissues [12,13], however, these results must be considered with caution due to possible artefacts of the quantification method used. Several methods and techniques can be used to measure mRNA levels. Northern blot analysis has traditionally been used, but more recently, real-time RT-PCR technology provides higher sensitivity and more accurate expression profiles [14,15]. In contrast to absolute quantification, relative quantification is not influenced by artefacts during sample preparation and it provides the means to detect small expression differences. However, this technique is more demanding than absolute quantification in the sense that it requires the selection of highly stable housekeeping (HK) genes to normalise the expression of the target gene. Although several studies on ovine gene expression have been reported using a single HK gene, the use of at least three stable HK genes is more suitable [16].

The aim of this work was to select and evaluate the stability of several ovine HK genes for relative expression analyses, and use them to test the hypothetical linkage between *PrP *gene expression and genetic susceptibility to scrapie.

## Results

### Primer optimisation and amplification specificity

Primer concentrations that generated the lowest Ct value and a sharp peak, but lacked non-specific fragments and primer-dimers were selected (Table 1). The analysis of melting temperatures, amplicon sizes and sequencing data demonstrated the specificity of the PCR reactions. The efficiency values obtained for the real-time PCR amplification of the six HK genes and *PrP *gene were near to 2. Efficiency values (E), slope values and correlation coefficient (R^2^) for each primer pair are shown in Table 1.

**Table 1:** Primers sequences and real-time PCR amplification parameters

Gene	Forward & reverse primers 5' → 3'	[C] ^a^	Amplicon size (bp)	Tm ^b ^(°C)	Slope	R^2c^	E ^d^
*ACTB*	ATGCCTCCTGCACCACCA GCATTTGCGGTGGACGAT	300	125	85	-3.597	0.999	1.897
*YWHAZ*	TGTAGGAGCCCGTAGGTCATCT TTCTCTCTGTATTCTCGAGCCATCT	100	102	79	-3.335	0.988	1.995
*RPL19*	CAACTCCCGCCAGCAGAT CCGGGAATGGACAGTCACA	200	76	83	-3.342	0.992	1.992
*GAPDH*	ATGCCTCCTGCACCACCA AGTCCCTCCACGATGCCAA	100	76	84	-3.485	0.991	1.936
*G6PDH*	TGACCTATGGCAACCGATACAA CCGCAAAAGACATCCAGGAT	300	76	81	-3.363	0.965	1.983
*SDHA*	CATCCACTACATGACGGAGCA ATCTTGCCATCTTCAGTTCTGCTA	200	90	82	-3.643	0.992	1.881
*PrP*	GCCAAAAACCAACATGAAGCAT TGCTCATGGCACTTCCCAG	300	95	83	-3.338	0.995	1.993

^a ^Primer concentrations in nM

^b ^Theoretical amplicon melting temperature calculated with Primer Express software (Applied Biosystems)

^c ^Correlation coefficient

^d ^PCR efficiency

### Selection of the optimal HK genes and normalisation of gene expression

The analysis of the expression of the HK genes in all six tissues in the 22 animals showed a pairwise variation above the cut off value (V_n/n+1_> 0.15) established by Vandesompele *et al*. [16]. This indicated the invalidity of using a common set of HK genes for all the tissues and therefore, the stability of the six HK genes was assessed within each tissue. The initial comparison of the M values for the six genes (Table 2) showed a large tissue-associated variation in the expression stability of some genes, which in some cases showed even opposite values. The stepwise exclusion of the less stable HK gene according to the geNorm application showed six different stability series, one for each tissue (Table 3), confirming the tissue-associated variation. For instance, the *G6PDH *gene showed high stability in cerebellum and ileum while it was the less stable gene in spleen. In addition, the frequently used HK gene *GAPDH *showed the smallest variation when the M value from each tissue was compared (Table 2) and it was selected for the normalisation in five of the six tissues analysed (Table 3), confirming its high stability. On the other hand, another traditionally used HK gene, *ACTB*, showed the second highest standard deviation among tissues in comparison to other HK genes.

**Table 2:** Expression stability values (M) of the six candidate HK genes

Tissue	*ACTB*	*YWHAZ*	*RPL19*	*SDHA*	*GAPDH*	*G6PDH*
Cerebrum	0.602	0.757	0.634	0.540	0.510	0.660
Cerebellum	0.477	0.480	0.452	0.518	0.473	0.457
Obex	0.534	0.493	0.593	0.541	0.451	0.479
Spleen	0.436	0.413	0.550	0.427	0.448	0.569
Mesenteric lymph node	0.556	0.691	0.542	0.525	0.502	0.624
Ileum	0.757	0.652	0.696	0.610	0.517	0.511

Mean	0.560	0.581	0.578	0.527	0.484	0.550
SD ^a^	0.113	0.137	0.084	0.059	0.030	0.081
CV (%)^b^	20.133	23.669	14.543	11.192	6.268	14.771

M values in this table were calculated for the six HK genes previous to the stepwise exclusion of the less stable HK gene.

^a ^Standard deviation

^b ^Coefficient of variation

**Table 3:** HK genes stability series for each tissue

Cerebrum	Cerebellum	Obex	Spleen	Mesenteric lymph node	Ileum
***GAPDH-SDHA***	***G6PDH-ACTB***	***GAPDH-YWHAZ***	***GAPDH-SDHA***	***SDHA-RPL19***	***GAPDH-G6PDH***
***ACTB***	***YWHAZ***	***G6PDH***	***ACTB***	***GAPDH***	***SDHA***
*G6PDH*	*RPL19*	*SDHA*	*YWHAZ*	*ACTB*	***YWHAZ***
*RPL19*	*GAPDH*	*ACTB*	*RPL19*	*G6PDH*	*RPL19*
*YWHAZ*	*SDHA*	*RPL19*	*G6PDH*	*YWHAZ*	*ACTB*

The stability series are shown from the most stable gene at the top to the least stable gene at the bottom ranked to their expression stability estimated using geNorm. The first two genes in each series cannot be ranked because of the required use of gene ratios for gene stability measurements. The HK genes required for a reliable normalisation of the target gene expression in each tissue are shown in bold.

### Normalised PrP gene expression analysis

**Figure 1:**
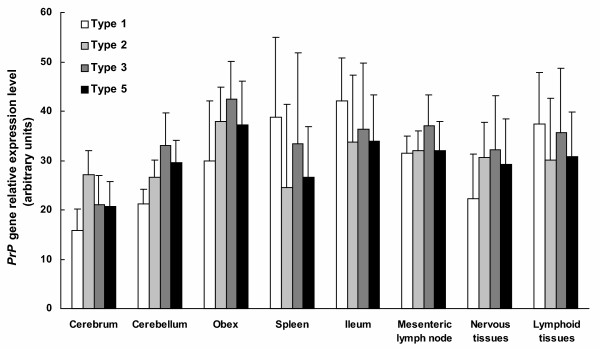
***PrP *gene expression levels in the different tissues of 22 sheep grouped in risk groups**. The *PrP *mRNA levels were obtained by relative quantification real-time RT-PCR analysis using the most stable HK genes within each tissue. Error bars represent standard deviation. Risk groups according to the NSP classification [7].

Classification of animals according to their risk group and results from the expression analyses are listed and graphically represented in Table 4 and Figure 1, respectively. A marked association between *PrP *mRNA level and the type of tissue (p < 0.0001) was found in the overall analysis including all risk levels. In this sense, the obex showed the highest expression level (38.05) followed by ileum (35.73), lymph node (33.51), spleen (29.99), cerebellum (28.89) and cerebrum (21.58). When the risk group effect was analysed, no significant association was found between it and the *PrP *mRNA levels. The model did not show any interaction between tissue type and risk group.

**Table 4:** Relative mRNA expression levels of *PrP *gene (arbitrary units)

Risk group		Cerebrum	Cerebellum	Obex	Spleen	Ileum	Mesenteric lymph node	Nervous tissues	Lymphoid tissues
									
	n ^a^	nPrP ^b ^(SD^c^)	nPrP (SD)	nPrP (SD)	nPrP (SD)	nPrP (SD)	nPrP (SD)	nPrP (SD)	nPrP (SD)
1	3	15.829 (4.279)	21.202 (3.052)	29.892 (12.223)	38.851 (16.115)	42.048 (8.800)	31.424 (3.496)	22.308 (9.057)	37.442 (10.470)
2	5	27.132 (4.830)	26.632 (3.495)	37.897 (6.930)	24.504 (16.953)	33.659 (13.712)	31.940 (4.088)	30.554 (7.267)	30.035 (12.551)
3	7	21.025 (5.869)	33.088 (6.515)	42.435 (7.598)	33.468 (18.426)	36.318 (13.434)	36.971 (6.320)	32.183 (11.001)	35.586 (13.054)
5	7	20.645 (5.092)	29.584 (4.439)	37.283 (8.811)	26.628 (10.323)	33.897 (9.467)	32.051 (5.895)	29.171 (9.247)	30.859 (8.903)

Total	22	21.584 (5.991)	28.885 (6.055)	38.054 (8.841)	29.989 (15.268)	35.725 (11.328)	33.506 (5.621)	29.508 (9.736)	33.073 (11.517)

Risk groups are listed from the most resistant one on the top to the most susceptible at the bottom according to the NSP classification [7]. Type 1, included three ARR/ARR animals; Type 2, included five ARR/ARQ animals; Type 3, included one ARH/ARH, one ARQ/ARH and five ARQ/ARQ animals; Type 5, included one ARH/VRQ, five ARQ/VRQ and one VRQ/VRQ animal.

^a ^Number of animals

^b ^Normalised *PrP *mRNA level

^c ^Standard deviation

When the expression values were considered within each tissue, several specific significant (p < 0.05) or marginally significant (p < 0.10) differences were found between pairwise comparisons. In cerebral samples, animals from Type 1 showed the lowest *PrP *gene expression value, followed by animals from Type 5, Type 3 and Type 2, but only the lower expression level in Type 1 than in Type 2 animals was marginally significant (p = 0.0700). In cerebellum samples, Type 1 showed the lowest expression value followed by Type 2, Type 5 and Type 3. Expression level in Type 1 was marginally lower than in Type 3 (p = 0.0668). In obex samples, Type 1 showed the lowest expression value followed by Type 5, Type 2 and Type 3. Again, expression level in Type 1 was marginally lower than in Type 3 (p = 0.0633). In spleen samples, Type 1 showed the highest expression value followed by Type 3, Type 5, and Type 2. Expression in Type 1 was significantly higher than in Type 2 (p = 0.0281) and in Type 5 (p = 0.0671), and in Type 2 lower than in Type 3 (p = 0.0937). In ileum samples Type 1 showed the highest expression value followed by Type 3, Type 5, and Type 2. In mesenteric lymph node samples Type 1 showed the lowest expression value followed by Type 2, Type 5, and Type 3. When data were grouped in nervous tissues (cerebrum, cerebellum and obex) *versus *lymphoid tissues (spleen, ileum and lymph node), the models showed a relationship between the *PrP *mRNA levels and the effect of tissue type (p = 0.0160). A statistical association was found for the interaction between tissue type and risk group (p = 0.0623). *PrP *mRNA level in lymphoid tissues was significantly higher than in nervous tissues (p = 0.0160). In nervous tissues, Type 3 showed the highest expression value followed by Type 2, Type 5, and Type 1. The expression found in Type 1 was lower than in Type 2 (p = 0.0496), Type 3 (p = 0.0174), and Type 5 (p = 0.0863). Regarding lymphoid tissues samples, Type 1 showed the highest expression value followed by Type 3, Type 5, and Type 2. Expression in Type 1 was higher than in Type 2 (p = 0.0840).

## Discussion

Real-time RT-PCR was chosen among several techniques available to measure the mRNA levels of *PrP *gene in six important tissues for the transmission and the development of scrapie. Real-time RT-PCR technology provides high sensitivity and accurate expression profiles [14,15] and in that approach, two basic protocols can be followed: absolute quantification and relative quantification. For gene expression studies, relative quantification is more suitable because the influence of unavoidable artefacts during sample preparation is taken into account. Several works on ovine gene expression have been carried out using the common practice of normalising with a single control gene like 18S rRNA, *GAPDH *or *ACTB *[17-19]. However, inter-individual variation of traditionally considered stable HK genes can be high enough to bias gene expression profiles when calculated using only one HK gene for normalisation. Therefore, the use of more than a single HK gene is recommended particularly to detect small expression differences more accurately. Thereby, the sensitivity of this approach depends on how well the HK genes are selected. Thus, in this study a robust method described by Vandesompele *et al*. [16] has been followed where the use of a minimum of three stable HK genes is required for an accurate normalisation of the target gene expression after assessing the stability of a given set of HK genes.

The expression stability observed in the six HK genes analysed in the present work varied with the tissue, and therefore, different sets of HK genes were necessary to normalise the *PrP *gene expression within each tissue. This variability of the HK genes stability among samples from a variety of sources is consistent with the literature [15,20-22] and reflects the existence of a tissue-specific metabolism and/or unknown tissue-specific factors. These findings clearly demonstrate that there is no single universal control gene for all tissues or cell types. Thereby, these inherent variations have to be taken into consideration and the stability of the HK genes needs to be studied in each scenario prior to any relative quantification study in order to obtain results as accurate as possible. *GAPDH *and *ACTB *have been traditionally considered invariable (equally expressed) genes and consequently, they have been widely used as single control genes for gene expression in many studies. However, the expression of these genes can vary from 7 to 23-fold depending on the cell type or tissue [21]. In our study, *GAPDH *gene showed the lowest variation among the panel of six tissues, whereas *ACTB *showed the penultimate worst score in variability. This is in agreement with the reported invalidity of *ACTB *gene for gene expression in ovine interstitial cells from heart valves [23]. Consistent with this variation and in order to improve the accuracy of our results, we normalised the *PrP *gene expression using the most stable sets of HK genes in each of the six tissues analysed.

In order to test the hypothesis of the linkage between *PrP *gene expression and *PrP *genotype-associated susceptibility, 22 Latxa sheep with different genotypes were analysed. Special care was taken with the statistical analysis because the number of samples available for our study was not too large. In this sense, a general linear model was used to control all the effects for which information was available in order to reduce risks of errors linked to repeated separate comparisons and to guarantee that no type α errors were committed. The results revealed statistically significant differences in the *PrP *gene expression among the panel of six tissues. The highest *PrP *mRNA expression level in CNS samples was found in obex followed by cerebellum and cerebrum. This circumstance might be translated into high levels of PrP^c ^and perhaps into high levels of PrP^Sc ^aggregates considering PrP^c ^as the substrate for conversion into the pathologic isoform. The different PrP^c ^content in the three tissues of the CNS would be in accordance with the spatiotemporal appearance of PrP^Sc ^aggregates and would also support the idea that the obex is the best source of material for the detection of PrP^Sc ^in TSE rapid tests analyses.

Factors inherent in the nature of the different tissues like transcripts stability or postranscriptional regulation of the *PrP *gene have also to be considered. In this context, some studies focused on *PrP *mRNA have shown that there are two *PrP *mRNA transcripts (2.1 and 4.6 kb mRNA) with a tissue-specific distribution, different stability rates and different efficiency of translation [24-26]. Moreover, a previous work revealed that the isoform profile and the abundance of the PrP^c ^in sheep were tissue-specific, showing lower PrP^c ^abundance in lymphoid tissues (three orders of magnitude) than in CNS tissues [27]. However, our results showed higher *PrP *transcripts in lymphoid tissues suggesting that a postranscriptional regulation of the *PrP *gene occurs in these tissues. Therefore, the comparison of *PrP *gene expression among tissues might be a very complex issue. When expression values from nervous tissues were grouped, a tendency towards increased mRNA expression levels of *PrP *gene and genetic susceptibility to scrapie was observed in CNS tissues. In these tissues, Type 1 animals showed the lowest expression levels and a gradual increase of *PrP *gene expression was found towards Type 3. Curiously, Type 3 animals showed more *PrP *mRNA levels than Type 5, however, no statistical differences were found. On the other hand, in spleen and ileum, Type 1 showed the highest expression levels. Inherent artefacts such as PCR inhibitors in spleen samples or the heterogeneous distribution of the immune system in ileum of different animals might have contributed to the high variability found in these samples.

In general, most of the differences found in this study were only marginally significant, and therefore, the results presented here cannot support the existence of a relationship between *PrP *mRNA levels and risk group. Interestingly, recent studies using a smaller number of samples have revealed *PrP *genotype-specific differences in PrP^c ^levels in mononuclear cells of peripheral ovine blood [28] and in the amount of PrP^Sc ^(but no PrP^c^) in experimentally infected sheep brain [29]. Therefore, if PrP^c ^synthesis were *PrP *genotype-dependent, this study would show that this association does not occur at the transcriptional level. However, an association at later stages, *i.e*. at the postranscriptional regulation level (including mRNA transport out of the nucleus, transcripts stability and regulation at the level of translation) cannot be excluded. Thus, since expression of genes is controlled at several steps, further studies applying different approaches are needed. In addition, the complexity of scrapie pathogenesis might also be influenced by other still unknown genes or strain-specific factors.

## Conclusion

The global overview of scrapie pathogenesis is quite complex, but being PrP^c ^the substrate for the conversion of the pathogenic form, *PrP *mRNA transcripts play an important role, and in this sense, the results on *PrP *gene expression presented here provide valuable baseline data for future studies. In any case, whatever the mechanism for susceptibility, this study showed that it is not related to the regulation of the *PrP *gene transcripts. On the other hand, the results on stability data of several HK genes reported in this study could prove very useful in other gene expression studies carried out in these ovine tissues. Future gene expression studies including a larger and more diverse (*i.e*. different breeds) set of samples would benefit from these data.

## Methods

### Sample selection

Twenty-two healthy sheep from Latxa breed were selected according to their susceptibility to scrapie [7] and the distribution of genotypes within the Latxa sheep population [30]. Hence, all the highly susceptible genotypes (Type 5) described in Latxa breed were included, along with those other genotypes present in more than 1.5% of the population. In any case, only the most prevalent genotypes were represented by more than one individual. Thus, three animals with ARR/ARR genotype, five ARQ/ARQ, one VRQ/VRQ, one ARH/ARH, five ARR/ARQ, five ARQ/VRQ, one ARQ/ARH and one animal with ARH/VRQ genotype were selected. *PrP *genotyping was performed by real-time PCR as previously described [30]. The age of the animals ranged from 3 to 12 years old. All the animals were negative to TSE by the PrP^Sc ^detection kit Platelia^®^BSE (Bio-Rad, Hercules, CA, USA) on an obex sample. Animals were sacrificed under controlled conditions and a sample from the same region of cerebrum (neocortex), cerebellum, obex, spleen, terminal ileum and mesenteric lymph node was aseptically taken from each animal. All tissues were frozen immediately at -80°C until RNA extraction was performed.

### RNA extraction and cDNA synthesis

Tissue samples were homogenised with a Ribolyzer (Hybaid, Ashford, UK) and total RNA was isolated using the RNeasy Protect Mini kit (Qiagen, Hilden, Germany). Total RNA was treated with DNase I (Ambion, Austin, TX, USA) to avoid genomic DNA amplification and first strand cDNA was synthesised using random hexamers and MultiScribe™ reverse transcriptase (Applied Biosystems, Foster City, CA, USA) according to manufacturer's instructions. In addition, the effectiveness of the DNase treatment was assessed in RT-negative samples. After reverse transcription, the same batch of diluted cDNA was subjected to real-time PCR to amplify six HK genes and the *PrP *gene.

### Primers design and optimisation

Six commonly used HK genes were selected to normalise the expression of the target gene *PrP*: β-actin (*ACTB*), tyrosine 3-monooxygenase (*YWHAZ*), ribosomal protein L19 (*RPL19*), glyceraldehyde-3-phosphate dehydrogenase (*GAPDH*), glucose-6-phosphate dehydrogenase (*G6PDH*) and succinate dehydrogenase (*SDHA*). Primers were designed to span one intron using Primer Express software (Applied Biosystems, Foster City, CA, USA) (Table 1). Primers for the *ACTB*, *GAPDH*, *RPL19*, *G6PDH *and *PrP *genes were designed from ovine sequences obtained from GenBank. For the *SDHA *and *YWHAZ *genes, ovine sequences were not available and therefore, multiple sequence alignments of these genes obtained from different animal species (*Bos taurus*,* Mus musculus*,* Rattus norvegicus *and *Homo sapiens*) were carried out using the program AlingX (Vector NTI 8.0 suite, Informax Inc., North Bethesda, MD, USA) to identify conserved regions for primer design. Four concentrations of primers (50 nM, 100 nM, 200 nM and 300 nM) were evaluated, and formation of primer-dimers was assessed by melting curve analysis. Thus, only those concentrations of primers which showed dimer-free reactions were used for the final analysis.

### Real-time RT-PCR

The same number of samples (22) for each tissue were analysed to prevent bias in the results. PCR reactions were set up with the automatic workstation Biomek 2000 (Beckman-Coulter, Fullerton, CA, USA) to minimise pippetting errors. Each sample was analysed in triplicate in a total reaction volume of 10 μl consisting of 10 ng of cDNA, 2xSYBRGreen buffer (Applied Biosystems, Foster City, CA, USA) and the required amount of forward and reverse primers (Table 1). Reactions were run on an ABI PRISM 7000 thermocycler (Applied Biosystems, Foster City, CA, USA) using the following cycling conditions: 95°C for 10 min and 40 cycles at 95°C for 15 s and 60°C for 1 min. For each experiment, a non-template reaction was included as negative control. The specificity of the PCR reactions was confirmed by melting curves analysis of the products as well as by size verification of the amplicons in a conventional agarose gel. In addition, PCR products from the HK genes and *PrP *gene were cloned into pCR^®^4-TOPO vector using TOPO TA Cloning^® ^kit (Invitrogen, CA, USA) and submitted to a commercial subcontractor for automatic dye-terminator cycle sequencing. The sequences of *SDHA *and *YWHAZ *genes were deposited in GenBank under accession nos. AY970969 and AY970970, respectively.

The threshold cycle values (Ct) were determined at the same fluorescence threshold line for each gene and the Ct value for each sample was obtained by calculating the arithmetic mean of the triplicate values when the standard deviation was lower than 0.16. Ct values were transformed into raw quantity values (Q) according to the following equation, Q = E ^(Min Ct-Sample Ct) ^(geNorm user manual, ), where "E" is the efficiency of the real-time PCR for each gene and "Min Ct" is the minimum Ct value for the samples analysed. E values were calculated for each gene from the given slope after running serial dilutions of cDNA and the following formula E= [10^(-1/slope)^] [31].

### Selection of the optimal HK genes and normalisation of PrP gene expression

The method described by Vandesompele *et al*. [16] was followed to assess the stability of the expression of the HK genes under study using the MS Excel application (geNorm 3.3). Briefly, this application calculates the expression stability measure (M) for the set of HK genes and selects the minimum number of HK genes needed for the normalisation. Thus, genes with the lowest M values have the most stable expression and following the stepwise exclusion of the less stable HK gene M values are re-calculated and the stability series is obtained. Once ranked, the minimum number of HK genes needed was calculated using a cut-off value of 0.15 for V_n/n+1 _[16]. The normalisation factor (NF) was then calculated as the geometric mean of their Q values.

Finally, the normalised expression level of the *PrP *gene (nPrP) was calculated as the ratio between the Q values of *PrP *gene amplification and the NF calculated for each sample.

### Statistical analysis

In order to compare among tissues, analysis of variance of the reference values (NF) was carried out with the GLM procedure of the SAS statistical package version 8 (SAS institute, Cary, NC, USA). Once the results of this model showed no significant differences among tissues, the PrP/NF ratios (nPrP) were transformed according to the formula arc sin √ (nPrPx100^-1^) as recommended for use of parametric tests on relative data. Then nPrP values along with all available independent variables (sex, age, tissue and genetic susceptibility) and their interactions were submitted to the GLM procedure of SAS statistical package version 8 (SAS institute, Cary, NC, USA). This analysis showed that sex and age had no significant effects and therefore, only tissue and genetic susceptibility and their interactions as independent variables for effects on nPrP were included in the final model. Genetic susceptibility was considered as risk levels [7]. Comparison of means was carried out using a Student's t test with the SAS statistical package version 8 (SAS institute, Cary, NC, USA).

## Competing interests

The author(s) declare that they have no competing interests.

## Authors' contributions

DG carried out the design of the study, the experimental work and drafting of the manuscript. RJ performed the statistical analysis and participated in the critical reading of the publication. AH participated in the design and coordination of the study and drafting of the manuscript. All authors read and approved the final manuscript.
