# Selection of ovine housekeeping genes for normalisation by real-time RT-PCR; analysis of *PrP *gene expression and genetic susceptibility to scrapie

**DOI:** 10.1186/1746-6148-2-26

**Published:** 2006-08-16

**Authors:** David Garcia-Crespo, Ramón A Juste, Ana Hurtado

**Affiliations:** 1 Department of Animal Health, Instituto Vasco de Investigación y Desarrollo Agrario (NEIKER), Berreaga, 1. 48160 Derio, Bizkaia, Spain

After the publication of this work [1], we became aware of the fact that in Table 1 there was a mistake in the sequence of the forward primer used for the amplification of the ACTB gene. The correct sequence (Table 1, column 2, line 1) should read as follows: 5'-CTGAGCGCAAGTACTCCGTGT-3'

We regret any inconvenience that this error might have caused.
